# A Systemic Review of Spinal Needles Broken During Neuraxial Anesthesia

**DOI:** 10.7759/cureus.40241

**Published:** 2023-06-11

**Authors:** Thamer H Alsharif, Amin G Gronfula, Shifaa Turkistani, Abdulkarim M Alshmlany, Zikra Sharourou, Hesham Aboueleneein, Fawaz Alhamied

**Affiliations:** 1 Neurosurgery, Royal College of Surgeons in Ireland, Dublin, IRL; 2 Orthopaedic Surgery, Royal College of Surgeons in Ireland, Dublin, IRL; 3 Medicine, Royal College of Surgeons in Ireland, Dublin, IRL; 4 General Practice, Royal College of Surgeons in Ireland, Dublin, IRL; 5 Neurosurgery, King Fahad Armed Forces Hospital, Jeddah, SAU; 6 Neurology, King Fahad Armed Forces Hospital, Jeddah, SAU

**Keywords:** neuraxial anesthesia complications, needle size, : pregnancy, broken needle, spinal anaesthesia

## Abstract

Needle breakage during spinal anesthesia occurs infrequently and represents a serious complication with potentially adverse effects. The objective of this systemic review was to look at the incidence, risk factors, and preventative measures for broken spinal needles. A search of the literature on PubMed, Web of Science, and Embase databases and a manual web search was performed, with no filters and up to April 2023 from inception. Out of the 43 potential studies, 23 were included. The search terms for the full article reading were broken needle, spinal anesthesia, humans, and post-operative, and the exclusion criteria were systematic reviews, conference presentations, and non-full articles. A review of the 23 studies (24 cases) suggests an association between specific risk factors such as obesity and needle size and breaks. Identifying the risks and complications of needle breaks could help physicians modify their practice and inform their patients of any increased risks applicable to them.

## Introduction and background

Numerous surgical procedures, particularly those in obstetrics, gynecology, and orthopedics, frequently involve spinal anesthesia. Although problems including post-dural puncture headache, nerve injuries, and epidural hematoma can happen. The breakage of a spinal needle within the patient's intrathecal space is one of the less common but potentially serious complications of spinal anesthesia [1,2]. Unfavorable outcomes from a broken spinal needle may include pain and infection [3]. As a result, it is critical to determine the risk factors for needle breakage during spinal anesthesia and to develop preventative measures.

Several case reports and series were published to deal with this rare complication and address the topic while looking at the incidence, risk factors, and ways to prevent broken needles while receiving spinal anesthesia. Besides pinpointing risk factors, some studies have additionally provided several measures that can lower the frequency of needle breakage; these include using a blunt-tipped needle, avoiding exerting too much effort while inserting the needle, and using a larger gauge needle [3-5].

Our objective in conducting this systematic review is to increase patient safety and outcomes by conducting a comprehensive and evidence-based assessment of the incidence, risk factors, and preventative measures for this potentially serious complication. We seek out and gather all relevant research on a particular currently available topic and analyze it.

## Review

Methods

The Preferred Reporting Items for Systematic Reviews and Meta-Analysis statement (PRISMA) criteria were followed in reporting this systematic review. Since the information was available to the general public, institutional review board approval was unnecessary.

Search Strategy 

**Figure 1:**
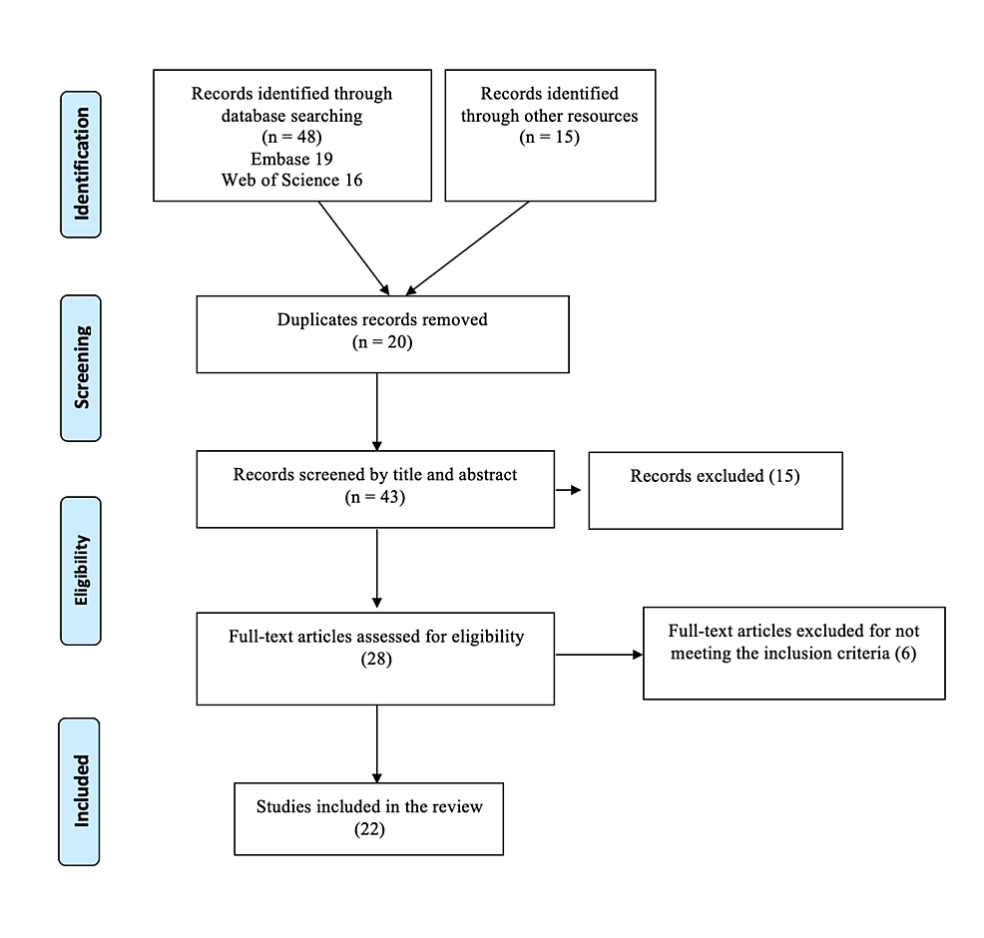
PRISMA Flow Diagram

A systematic search of databases (PubMed, Web of Science, and Embase) was conducted from their inception to April 2023. No filters were applied, such as language, publication country, or type of article, including abstracts and posters. The bibliographic references of the publications were manually examined to find any additional acceptable studies that might be included. An EndNote library was created by downloading the available results. The entire search strategy is displayed in the PRISMA diagram (Figure 1).

Study Selection and Data Extraction

EndNote 20 was used to import all the chosen studies, and duplicates were found and removed. The two reviewers, T.H. and A.G., looked at the titles and abstracts of the remaining studies. The full text was critically evaluated against the inclusion and exclusion criteria for the final selection of articles. Any inconsistencies were resolved by consulting a third reviewer (H.A.). The following eligibility criteria were used to select studies for inclusion: 1) case report and series, 2) retrospective studies, and 3) studies that included broken needles during spinal anesthesia. Observational studies and systematic reviews were all ruled out. Two investigators independently extracted data from the selected studies. Data collected included authors, year, title, gender, age, diagnosis, surgery, symptoms, and follow-up.

Results

Search Overview

An electronic search of the literature was identified using the search strategy of 48 articles (19 Embase, 16 Web of Science, and 13 PubMed), in addition to 15 articles added through a manual web search. There were 20 duplicates among the 63 studies. The titles and abstracts of the remaining 43 articles were screened, and 28 articles were examined for full-text screening. In the final analysis, we included 22 articles.

Study Characteristics

The review included 23 cases, whereby 74% of the subjects were female, and 26% were male. These studies were published up until 2023 and were carried out across various countries, including the United States, India, Denmark, the United Kingdom, France, Burkina Faso, Brazil, the Netherlands, Germany, Korea, Indonesia, China, Taiwan, and Saudi Arabia. The participants ranged from 18 to 71 years, with a mean age of 33.05. Pregnancy was the most common diagnosis, accounting for 16 cases. While the patients exhibited a significant range in body mass index, with a higher overall tendency (more details can be viewed in Table 1).

**Table 1:** Characteristics of included studies

Author	Country	Diagnosis	Weight (BMI)	Type of needle	Symptoms
Thomsen AF et al. [6]	Denmark	RA	86 kg	25 gauge Whitacre	No neurological deficits
Shah SJ et al. [7]	USA	Pregnancy	61.1 kg	24 gauge Whitacre	No neurological deficits
Mehta M et al. [8]	India	Pregnancy	N/A	N/A	N/A
Martinello C et al. [9]	USA	Pregnancy	48.2 kg/m^2^	25 gauge Whitacre	No neurological deficits
Kaboré RAF et al. [10]	Burkina Faso	Pregnancy	42 kg/m^2^	25 gauge Whitacre	No neurological deficits
Greenway MW et al. [11]	UK	Pregnancy	50 kg/m^2^	27 gauge Whitcare	No neurological deficits
Gentili ME et al. [12]	France	N/A	N/A	N/A	No neurological deficits
Eng M et al. [13]	USA	N/A	N/A	25 gauge Whitacre	N/A
Cruvinel MGC et al. [14]	Brazil	Hypertension and Renal failure	29.38 kg/m^2^	27 gauge Whitacre	N/A
Abou-Shameh MA et al. [15]	Netherland	Pregnancy	110 kg	27 gauge Whitacre	No neurological deficits
Rieg AD et al. [16]	Germany	Pregnancy	31 kg/m^2^	28 gauge Whitacre	Motor failures and paraesthesia
You JW et al. [17]	Korea	Spinal stenosis	24.83 kg/m^2^	N/A	Right leg & back pain
Arie Utariani et al. [18]	Indonesia	Pregnancy	49.9 kg/m^2^	25 gauge Whitacre	No any neurological deficits
Arie Utariani et al. [18]	Indonesia	Pregnancy	32.42 kg/m^2^	26 gauge spinocain	No any neurological deficits
Thamer Alsharif et al. [19]	Saudi Arabia	Pregnancy	N/A	N/A	Lower back pain
Dunn et al. [20]	USA	Pregnancy	39.4 kg/m^2^	18 gauge Tuohy	No any neurological deficits
Hershan et al. [21]	USA	Pregnancy	180 Kg	17 gauge Touhy	No any neurological deficits
Lonnée et al. [22]	China	Pregnancy	30 Kg/m^2^	27 gauge Whitacre	Lower back pain
Wendling et al. [23]	USA	Pregnancy	37.4 kg/m^2^	27 gauge Whitacre	N/A
The J et al. [24]	USA	Pregnancy	115 kg	27 gauge Whitacre	N/A
Kwan WF et al. [25]	USA	Pregnancy	32.74 kg/m^2^	25 gauge Whitacre	N/A
Chaney MA et al. [26]	USA	Peripheral Arterial Disease	N/A	24 gauge Sprotte	N/A
Sharma P et al. [27]	India	Knee contracture	N/A	23 gauge Quincke	N/A

Diagnosis and Symptoms Observed Following the Breakage of the Needle

Table 2 shows that the findings revealed a diverse range of diagnoses among the cases examined. Numerous pregnancy occasions were noticed, demonstrating its prevalence in the study population. In addition, cases of rheumatoid arthritis, hypertension, renal failure, spinal stenosis, peripheral arterial disease, and broken acupuncture needles were identified. Notably, some cases were given the status "N/A," indicating no diagnosis was mentioned in the study.

**Table 2:** Diagnosis and Gender distribution

Diagnosis	Female	Male	Grand Total
Pregnancy	16		16
Hypertension and Renal failure		1	1
Knee contracture		1	1
Peripheral Arterial Disease		1	1
RA		1	1
Spinal stenosis		1	1
N/A	1	1	2
Grand Total	17	6	23

The study findings regarding the associated symptoms post needle breakage of the patient showed that no neurological symptoms were identified in pregnant patients. In the case of rheumatoid arthritis (RA), no symptoms suggested neurological deficits. There were no specific symptoms listed for cases of renal failure or hypertension. One patient with spinal stenosis experienced side effects of severe pain in the right leg and back. No particular side effects were accounted for in people determined to have peripheral arterial disease. Given the limited number of cases, these symptoms provide fragments of insight into some of the symptoms following the breakage of spinal anesthesia.

Risk Factors

Obesity: Considering the association between high weight and BMI with an increased risk of needle breakage during spinal anesthesia, several of the results in the studies fall within the higher weight/BMI categories. A few high-weight measurements among the results: 110, 115, and 180 kilograms. Because higher BMI is known to make needle insertion more difficult and may necessitate additional precautions, they raise the possibility of needle breakage during spinal anesthesia [28]. On the other hand, there are also somewhat lower weight measurements: 61.1 kg and 86 kg. In addition, the results from BMI 37.4 kg/m^2^ to BMI 50 kg/m^2^. Individuals in these categories may have a higher proportion of body fat or a larger body mass, which can pose challenges during needle insertion. While the exact BMI thresholds for increased risk of needle breakage are not provided, it is generally recognized that higher BMI values, particularly in the obese range, can make it more difficult to locate the intrathecal space accurately and insert the needle safely. Factors such as increased subcutaneous fat changes in tissue planes and altered anatomical landmarks in individuals with higher BMI can contribute to technical difficulties during the procedure [29]. On the other hand, the results falling within the lower BMI categories, such as BMI 22.98 kg/m^2^ and BMI 24.83 kg/m^2^, may indicate a relatively lower risk of needle breakage. Individuals with lower BMI values typically have less body fat and may present fewer challenges during needle insertion [29].

Needle size: The type of needle used in spinal anesthesia. Different needle designs and gauges have their own advantages and drawbacks. Whitacre needles, like the 24, 25, 27, and 28 gauge choices, are usually utilized for spinal anesthesia [30]. The pencil-point shape of these needles may reduce the likelihood of post-dural puncture headache (a potential complication) [31]. Quincke, a needle with a gauge of 23 and 26, is another option. A spinal anesthetic can also be performed with Sprotte needles, including the 18-gauge and 24-gauge options. With a side hole near the tip, these needles have a unique design that allows for a more controlled flow of local anesthesia. Tuohy needles, which come in gauges 18 and 17, are typically used for epidural anesthesia [32]. These needles have a bent tip and are normally utilized for catheter position. It is essential to remember that the kind of needle used may be determined by various factors, such as the clinician's experience, the patient's characteristics, and the procedure's particular requirements. A qualified healthcare professional should conduct a thorough evaluation of the patient and the requirements of the procedure before selecting a type of needle. Table 3 shows the needle type distribution in the cases.

**Table 3:** Distribution of Needle Types used in administering the spinal anesthesia

Type of needle	Count of Type of needle
Whitacre	14
Sprotte	2
Quincke	2
Tuohy	1
N/A	4
Grand Total	23

Discussion

A few factors account for most needle breakage incidents among the articles analyzed. Here, we discuss the main factors and corresponding preventative measures.

Difficult Anatomical Landmarks

Difficulties in finding and palpating anatomical landmarks were associated with a higher risk of needle fracture. This was more frequently demonstrated in patients with increased body habitus, such as parturients and patients with obesity (BMI > 30) [33]. A few characteristics in pregnancy could make palpation of the lumbar landmarks difficult when undergoing spinal punctures, such as hyperlordosis, progressive pelvic rotation over the long axis of the spinal column, oedema, and weight gain [34]. The increased technical difficulty in performing a spinal block is associated with the poor quality of anatomical landmarks [35]. We postulate that in these cases, incorrect needle placement may be the underlying cause of the higher number of spinal needle fracture incidents in parturient and obese patients. Considering that the prevalence of obesity has been increasing in the last few decades, along with the fact that spinal anesthesia is commonly performed in obstetric settings, these become crucial risk factors to address when performing spinal punctures in such a population to decrease the risk of unwanted consequences [36,37].

Small-Gauge Needles

Small gauge needles are frequently used in spinal anesthesia due to their advantage in reducing post-dural headache, which is one of the commonest complications of spinal anesthesia. However, this review highlights a rarer but potentially more dangerous complication of spinal anesthesia. Eighteen out of all the reviewed cases reported using needles with a gauge of 25 or smaller. Our finding showed that small gauge needles may lead to a higher risk of needle fracture. This could be explained by the in vitro study conducted by Sitzman et al., which reported that a smaller needle gauge correlated with a higher deflection magnitude [38,39]. However, we must remember that smaller gauge needles are more commonly utilized than larger gauge needles. To our knowledge, this is the first systematic review to show an association between the spinal needle gauge size and the risk of needle fracture. However, more studies are needed to properly assess this correlation's mechanism.

Pre-puncture ultrasound guidance is suggested to counteract the dilemma of a difficult spinal puncture with the existing challenge of the abovementioned risk factors. In a randomized controlled trial, Sahin et al. demonstrated a reduction in the time of spinal procedures in patients who underwent pre-puncture ultrasound guidance. In addition, they showed that its use in obese pregnant women significantly increased the first puncture attempt success rate [35].

The limitation of our review is that other risk factors were not possible to include in our analysis as they were inconsistently mentioned throughout the articles. Some of these factors include the physician's experience level, patient movement, multiple needle insertion attempts, and specific needle insertion.

## Conclusions

The breaking of a SA needle is a severe and rare incident of multifactorial causes. Our aim in this systematic review is to assess the incidence, risk factors, and preventative measures for this to improve patient safety and outcomes. The findings provide insights into the symptoms associated with different diagnoses and how difficult anatomical landmarks can lead to a higher risk of needle fracture. In addition, we focused on multiple risk factors, including high MBI and small gauge needles. However, more studies are needed to properly assess this correlation's mechanism and investigate further risk factors.
